# Synthesis, Characterization, Antioxidant Activity, Antibacterial Activity, and Cytotoxicity of Quaternized Inulin Derivatives Bearing Aromatic Amides

**DOI:** 10.3390/antiox14091091

**Published:** 2025-09-06

**Authors:** Yuan Chen, Yingqi Mi, Zhanyong Guo, Hongwu Zhang

**Affiliations:** 1 School of Materials Science and Engineering, Ludong University, Yantai 264025, China; chenyuan@ldu.edu.cn; 2 Yantai Institute of Coastal Zone Research, Chinese Academy of Sciences, Yantai 264003, China; yqmi@yic.ac.cn; 3 School of Marine Science and Fisheries, Jiangsu Ocean University, Lianyungang 222005, China

**Keywords:** antioxidant activity, antibacterial activity, cytotoxicity

## Abstract

In this study, a total of 12 new quaternized inulin (QIL) derivatives bearing aromatic amides were synthesized according to the ion exchange method. All the derivatives exhibited higher antioxidant activities in scavenging hydroxyl radicals, DPPH radicals, and superoxide radicals compared to pure inulin. Most of the derivatives could fully eliminate hydroxyl radicals at 1.6 mg/mL. Meanwhile, QIL derivatives exhibited increased antibacterial activity against *Escherichia coli* and *Staphylococcus aureus* compared to unmodified inulin. The structure–function relationship of the synthesized derivatives was discussed. Moreover, assays conducted with L929 cells (mouse fibroblasts) by the cell counting kit-8 (CCK-8) method did not show toxicities for the derivatives. Thus, the derivatives show promise for biomedical materials, functional foods, and pharmaceutical applications because they combine excellent antioxidant and antibacterial activities without exhibiting cytotoxicity.

## 1. Introduction

Antioxidant and antibacterial activities are crucial in biomedicine, with widespread applications in pharmaceuticals, functional foods, and biomedical materials. These activities help reduce oxidative damage, promote tissue repair, and prevent infections, playing a vital role in maintaining health and supporting therapeutic interventions [1]. Given the escalating challenges of antibiotic resistance and chronic diseases, the research and development of agents with dual antioxidant and antibacterial functions represent a critical direction for advancing modern medicine [2,3].

Inulin is a linear polymer whose basic structural unit is *β*-*D*-(2→1)-fructofuranose, typically ending with an *α*-*D*-(1→2)-glucopyranosyl unit [4]. The degree of polymerization (DP) of inulin ranges from 2 to 100, which is influenced by factors such as the plant source, the harvest time, the conditions under which inulin is processed, and the storage conditions [5]. It has been proved that inulin is helpful for improving intestinal microenvironment, improving intestinal immunity, promoting mineral absorption, regulating blood glucose level, and relieving constipation [6]. Therefore, more and more people are incorporating inulin into their daily diet. In addition, a number of inulin derivatives have been designed to improve the bioactivities of inulin, thus meet its application in food, cosmetics, pharmaceutical, and biomaterial fields [7,8]. Previous research demonstrated that the modification of inulin with various functional groups, including amino-pyridine [9], Schiff bases [10], coumarin [11], 1,2,3-triazole [12], and phosphate moieties [13], led to a significant enhancement in its antioxidant properties compared to unmodified inulin. Recently, Tan et al. [14] found that increasing the content of caffeic acid-grafted inulin significantly enhanced the DPPH radical scavenging ability, reducing power, and antibacterial activity of the active packaging films. Consequently, the film effectively delayed the aging of strawberries and preserved their postharvest quality.

Cationic polysaccharides have significantly advanced their industrial applications due to their biodegradability, low cost, and low toxicity. This modification improves the water solubility of polysaccharides, enhances their flocculation properties, and significantly increases their antibacterial activity [15]. The integration of cationic polysaccharides with anionic compounds can produce hydrogel-like structures, thereby broadening the applications of the former [16]. Cationic polysaccharides have been widely developed in diverse areas such as water treatment, food, papermaking, cosmetic, chemical, biomaterial, and petroleum industries [17,18,19]. The reaction frequently employed for the cationization of polysaccharides involves etherification using the (3-chloro-2-hydroxypropyl) trimethylammonium chloride (CHPTAC), and the products were also named as quaternized polysaccharides. Rahul et al. [20] successfully synthesized quaternized inulin (QIL) using base mediated cationization protocol, and the product exhibited high removal efficiency of 88.61% within 15 min against algal. Amjadi et al. [21] took QIL as a new surface decoration hydrocolloid and significantly improved the stability and bioavailability of liposomal nanocarriers. However, current research on further derivatization of QIL is inadequate, especially considering its potential applications. To the best of our knowledge, no studies have been conducted on the biological activity of QIL, despite its significant potential for applications in fields such as food, cosmetics, pharmaceuticals, biomaterials, etc.

The amide functional group (-CONR1R2) is one of the most prevalent functional groups found in various biomolecules, including proteins, peptides, and DNA, as well as in organic compounds and bioactive natural products such as paclitaxel and penicillin. Amides also serve as important industrial raw materials and synthetic intermediates and have been extensively studied and utilized across diverse fields, including organic chemistry, materials science, biology, and pharmaceuticals [22,23]. Aromatic amides, in particular, are of significant interest in medicinal and organic chemistry due to their broad spectrum of biological properties, such as antimicrobial, insecticide, anti-inflammatory and anti-cell death/apoptosis, antiviral, and antioxidant [6,24,25].

Based on the description above, we hypothesize that further research focused on modifying QIL with aromatic amides is of great importance, particularly for advancing its potential applications in biomedical materials, functional foods, and pharmaceuticals. Therefore, it is crucial to develop highly efficient strategies for incorporating aromatic amides into QIL. Furthermore, it is essential to investigate the antioxidant, antibacterial, and cytotoxic properties of these derivatives, as there is a significant gap in the current literature addressing this area of research.

In this study, chemical synthesis and structural characterization were conducted in the first step. Then, the antioxidant activities, antibacterial activities, and biocompatibilities of these derivatives were evaluated. The results will establish a theoretical foundation for the application of inulin derivatives as natural antioxidants in food preservation and pharmaceutical products.

## 2. Materials and Methods

### 2.1. Materials

#### 2.1.1. Reagents

Inulin (derived from chicory), with a molecular weight ranging from 2000 to 4000, was purchased from Xi’an Bai-Chuan Biotechnology Co., Ltd. (Xi’an, China) The compound (3-chloro-2-hydroxypropyl) trimethylammonium chloride (CHPTAC, 60 wt% aqueous solution), safranine T, Vitamin C, azithromycin, and various amine regents (including aniline, 2-fluoroaniline, 3,4-difluoroaniline, 3-chloroaniline, 4-chloroaniline, 3,4-dichloroaniline, 2-chlorobenzylamine, 2-bromoaniline, 2-aminopyridine, 3-aminopyridine, 4-aminopyridine, and 2-aminothiazole) were procured from Sigma-Aldrich Chemical Co., Ltd. (Shanghai, China). Additionally, sodium hydroxide, succinic acid, carbonyldiimidazole (CDI), dimethyl sulfoxide (DMSO), ethanol, 2,2-diphenyl-1-picrylhydrazyl (DPPH), sodium phosphate dibasic dodecahydrate, sodium phosphate monobasic dihydrate, disodium ethylenediaminetetraacetate (EDTA-2Na), iron sulfate heptahydrate, hydrogen peroxide, hydrochloric acid, nitrotetrazolium blue chloride (NBT), tris(hydroxymethyl)aminomethane (Tris), phenazine methosulfate (PMS), and nicotinamide adenine dinucleotide (NADH), were obtained from Sinopharm Chemical Reagent Co., Ltd. (Shanghai, China). Roswell Park Memorial Institute (RPMI)-1640 was purchased from Thermo Fisher Scientific Co., Ltd. (Shanghai, China). All chemical reagents in this study were of analytical-grade purity, and no further purification was deemed necessary.

#### 2.1.2. Bacterial Strains and Cell Line

The Gram-positive bacterium *Staphylococcus aureus* and the Gram-negative bacterium *Escherichia coli* were provided by the Shanghai Institute of Biochemistry and Cell Biology, Chinese Academy of Sciences. The mouse fibroblast cell line (L929) was also supplied by the same institute.

### 2.2. Chemical Synthesis

In the synthesis process, QIL was first synthesized by the etherification of free hydroxyl groups of inulin with CHPTAC. It is a cationic polymer with positive charges that can electrostatically interact with anionic compounds. On the other hand, amine was first reacted with succinic acid under the catalytic action of carbonyldiimidazole (CDI). Subsequently, the unreacted carboxylic groups were treated with NaOH to convert them into their ionic form, which then underwent an ion exchange reaction with QIL inside the dialysis bag. The unreacted ions were removed through the dialysis bag to obtain the final products.

#### 2.2.1. Preparation of Anionic Aromatic Amides

Succinic acid (1.18 g, 10 mmol) was dissolved in 10 mL of DMSO. CDI (1.62 g, 10 mmol) was then added to the solution, and the mixture was continuously stirred and reacted at 60 °C for 12 h under a nitrogen atmosphere. Subsequently, 10 mmol of amine solutions prepared in 5 mL of DMSO were added dropwise and allowed to react for an additional 12 h. The amine regents involved in the reaction included aniline, 3-chloroaniline, 2-fluoroaniline, 3,4-difluoroaniline, 3,4-dichloroaniline, 2-chlorobenzylamine, 2-bromoaniline, 4-chloroaniline, 2-aminopyridine, 3-aminopyridine, 4-aminopyridine, and 2-aminothiazole. Finally, the pH of the reaction mixture was adjusted to 8 using 2 M NaOH to obtain the anionic aromatic amide.

#### 2.2.2. Preparation of QIL

The preparation of QIL followed the procedure documented in the literature with slight modifications [20]. Briefly, 10 mmol of inulin was dissolved in an appropriate volume of deionized water. Subsequently, 15 mL of a 40 wt% sodium hydroxide solution was tardily added to activate the hydroxyl groups of inulin while stirring at 60 °C for 4 h. Afterward, 40 mL of CHPTAC was gradually added. The solution was continuously stirred at 60 °C for 10 h. The mixture was then cooled to room temperature (RT) and dialyzed in ultrapure water for 48 h using a dialysis tube with a molecular weight cutoff (MWCO) of 500 Da. Finally, the solvent was removed by freeze-drying at −60 °C under vacuum to arrive QIL.

#### 2.2.3. Preparation of QIL Derivatives Bearing Aromatic Amide

**Scheme 1:**
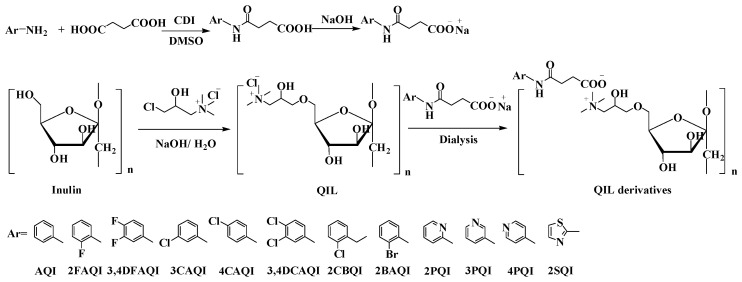
Synthetic route of the QIL derivatives.

0.6 g (2 mmol) of QIL, dissolved in 15 mL of deionized water, was mixed with the prefabricated anionic aromatic amide. After stirring at 30 °C for 12 h, the solution was dialyzed (MWCO = 500 Da) against ultrapure water for 48 h to remove unreacted reagents. Finally, QIL derivatives bearing aromatic amides were obtained by freeze-drying. The synthesis process of QIL derivatives is delineated in Scheme 1.

### 2.3. Characterization of Native Inulin and Inulin Derivatives

#### 2.3.1. FTIR (Fourier-Transform Infrared) Spectroscopy

The FTIR spectra of inulin, QIL, and QIL derivatives were recorded in the wavenumber range of 4000–400 cm^−1^ using a Nicolet iS50 infrared spectrometer from Thermo Fisher Scientific Co., Ltd., Shanghai, China. Sample preparation was performed using potassium bromide pelletization method for transmission measurements. The instrument operated in transmittance mode with a spectral resolution of 4.0 cm^−1^, acquiring 32 scans per sample under ambient temperature conditions.

#### 2.3.2. ^1^H NMR (Nuclear Magnetic Resonance) Spectroscopy

Samples (inulin, QIL, and QIL derivatives) were dissolved in D_2_O and subsequently analyzed using at a 500 MHz Bruker AVIII spectrometer (Switzerland, supplied by Bruker Tech. and Serv. Co., Ltd., Beijing, China), with tetramethyl silane (TMS) serving as the internal standard.

#### 2.3.3. Degrees of Substitution (DS)

The calculation of DS was based on the ^1^H NMR spectroscopy in accordance with established literature methods [26]. The results were derived from the ratio of the integral values associated with hydrogen protons on phenyl, pyridyl, and thiazolyl groups to those of the hydrogen protons (*H4*) present in the anhydrofructose units (AFU) of the inulin backbone. The equation is shown as follows:

(1) $$DS\;(\%)\;=\;\frac{I_{\mathrm{Hd},\mathrm{aryl}}}{I_{H4,\mathrm{AFU}}}\times 100$$

where $I_{\mathrm{Hd},\mathrm{aryl}}$ means the integral value of hydrogen protons on phenyl, pyridyl, and thiazolyl, and ${\;I}_{H4,\mathrm{AFU}}$ means the integral value of H4 present in the AFU of the inulin backbone.

### 2.4. Antioxidant Assays

#### 2.4.1. Hydroxyl Radical Scavenging Assay

The hydroxyl radical scavenging activity was assessed refer to the previous literature. [27]. Briefly, sample solutions at concentrations ranging from 0.45 to 7.2 mg mL^−1^ (1 mL each) were mixed with EDTA-Fe^2+^ (0.5 mL, 200 µmol/L), potassium phosphate buffer (1 mL, 150 mmol/L, pH 7.4), safranine T (1 mL, 0.23 µmol/L), and hydrogen peroxide (1 mL, 60 µmol/L). The mixture was incubated at 37 °C in the dark for 30 min before measuring absorbance at 520 nm using a 96-well plate reader. Vitamin C (VC) served as the positive control. Each assay was performed in triplicate, and scavenging activity was calculated according to the following equation.

(2) $$\mathrm{Scavenging}\;\mathrm{rate}\;(\%)\;=\;(\frac{A_{\mathrm{sample}\;517\;\mathrm{nm}}-A_{\mathrm{control}\;517\;\mathrm{nm}}}{A_{\mathrm{blank}\;517\;\mathrm{nm}}})\times 100$$

where

$A_{\mathrm{sample}\;520\;\mathrm{nm}}$: Absorbance of the sample reaction mixture.

$A_{\mathrm{blank}\;520\;\mathrm{nm}}$: Absorbance of the reagent blank (distilled water substituted for sample solution).

$A_{\mathrm{control}\;520\;\mathrm{nm}}$: Absorbance of the negative control (distilled water substituted for sample solution and phosphate buffer substituted for hydrogen peroxide solution).

#### 2.4.2. DPPH Radical Scavenging Assay

The DPPH radical scavenging activity was determined according to a published method [26] with slight modifications. Briefly, sample solutions (1 mL) at concentrations ranging from 0.3 to 4.8 mg mL^−1^ were mixed with DPPH solution (2 mL, 180 µmol L^−1^ in ethanol). After incubation at RT in the dark for 20 min, absorbance was measured at 517 nm using a 96-well plate. VC served as the positive control. Assays were performed in triplicate, and scavenging activity was calculated using the following equation:

(3) $$\mathrm{Scavenging}\;\mathrm{rate}\;(\%)\;=\;1-(\frac{A_{\mathrm{sample}\;517\;\mathrm{nm}}-A_{\mathrm{control}\;517\;\mathrm{nm}}}{A_{\mathrm{blank}\;517\;\mathrm{nm}}})\times 100$$

where

$A_{\mathrm{sample}\;517\;\mathrm{nm}}$: Absorbance of the sample reaction mixture.

$A_{\mathrm{blank}\;517\;\mathrm{nm}}$: Absorbance of the reagent blank (distilled water substituted for sample solution).

$A_{\mathrm{control}\;517\;\mathrm{nm}}$: Absorbance of the negative control (ethanol substituted for DPPH solution).

#### 2.4.3. Superoxide Radical Scavenging Assay

Superoxide radical scavenging activity was assessed according to the published literature [26]. Sample solutions (1.5 mL) at concentrations ranging from 0.2 to 3.2 mg mL^−1^ were mixed with NADH (0.5 mL, 456 μmol/L), NBT (0.5 mL, 300 μmol/L), and PMS in Tris-HCl buffer (0.5 mL, 60 μmol/L). The mixture was incubated at RT in the dark for 5 min before measuring absorbance at 560 nm in a 96-well plate. VC served as the positive control. Assays were performed in triplicate, and scavenging rate (%) was calculated by the following equation:

(4) $$\mathrm{Scavenging}\;\mathrm{rate}\;(\%)\;=\;(1-\frac{A_{\mathrm{sample}\;560\;\mathrm{nm}}-A_{\mathrm{control}\;560\;\mathrm{nm}}}{A_{\mathrm{blank}\;560\;\mathrm{nm}}})\times 100$$

where

$A_{\mathrm{sample}\;560\;\mathrm{nm}}$: Absorbance of the sample reaction mixture.

$A_{\mathrm{blank}\;560\;\mathrm{nm}}$: Absorbance of the reagent blank (distilled water substituted for sample solution).

$A_{\mathrm{control}\;560\;\mathrm{nm}}$: Absorbance of the negative control (distilled water substituted for NADH solution).

### 2.5. Antibacterial Assay

#### 2.5.1. Preparation of Sample Solutions

Inulin, QIL, and QIL derivatives were dissolved in sterile water to prepare sample solutions with a concentration of 32 mg/mL. Then, 100 μL of each sample solutions were two-fold serially diluted 11 times in a 96-well plate.

#### 2.5.2. Minimum Inhibitory Concentration (MIC) and Minimum Biocidal Concentration (MBC) Assay

The antibacterial activity of inulin, QIL, and QIL derivatives against two distinct bacterial strains, including the Gram-positive bacterium *Staphylococcus aureus* and the Gram-negative bacterium *Escherichia coli*, was measured as described in the literature with slight modifications [28]. The result was presented in the form of MIC and MBC. At the beginning, the bacterial colony was inoculated in 20 mL of liquid medium (RPMI-1640) and continuously shaken at 37 °C until the exponential growth phase. The suspensions were then diluted to a concentration of 10^5^–10^6^ cells/mL with freshly sterilized liquid medium. Following this, 100 μL of the bacterial suspension was transferred into each well containing the pre-configured sample solutions and incubated at 37 °C for 20 h. The MIC refers to the lowest concentration of the experimental samples at which visible bacterial growth is completely inhibited. Following the MIC assay, 100 μL of mixed solutions showing no visible bacterial growth were evenly coated on agar plates to determine the MBC. The MBC is defined as the lowest concentration of the experimental samples that results in a 99.9% reduction in viable bacteria after incubation at 37 °C for 24 h. Azithromycin and sterile water were separately used as a positive and blank control instead of sample.

### 2.6. Cytotoxicity Assay

As far as we know, there has not been a comprehensive study on the biocompatibility of QIL and its derivatives. Therefore, the CCK-8 assay was employed to evaluate the viability of L929 cells (mouse fibroblasts) exposed to the experimental samples: inulin, QIL, and its derivatives. The cytotoxicity assay was conducted in accordance with previous studies [29]. Initially, revived L929 cells were suspended in 5 mL of RPIM medium and incubated in a CO_2_ incubator. After being passaged 2 times, the cell suspension was diluted 8 times, and transferred into 96-well plates, with 100 μL per well. When the cells were incubated to about 50%, the original culture medium was replaced with the prepared sample solutions to achieve final sample concentrations of 1000, 500, 100, 10, and 1 μg/mL. After 24 h of incubation, 10 μL of CCK-8 solution was added to each well, and the incubation continued for another 24 h. Finally, the absorbance at 450 nm was measured using a microplate reader (DNM-9602G, Thermo Multiskan Ascent, Waltham, MA, USA). Cell viability was calculated by the following equation:

(5) $$\mathrm{Cell}\;\mathrm{viability}\;(\%)\;=\;\frac{A_{\mathrm{sample}}-A_{\mathrm{blank}}}{A_{\mathrm{control}}-A_{\mathrm{blank}}}\times 100$$

where

$A_{\mathrm{sample}}$: the absorbance of the sample group (containing cells, CCK-8 solution, and sample solution).

$A_{\mathrm{blank}}$: the absorbance of the blank group (containing RPMI-1640 medium and CCK-8 solution).

$A_{\mathrm{control}}$: the absorbance of the control group (containing cells and CCK-8 solution).

### 2.7. Statistical Analysis

The results are presented as the means ± standard deviations (SD, *n* = 3), derived from a minimum of three parallel experiments. A *p*-value < 0.05 indicated statistical significance.

## 3. Results

### 3.1. Characterization of Native Inulin, QIL, and QIL Derivatives

**Figure 1:**
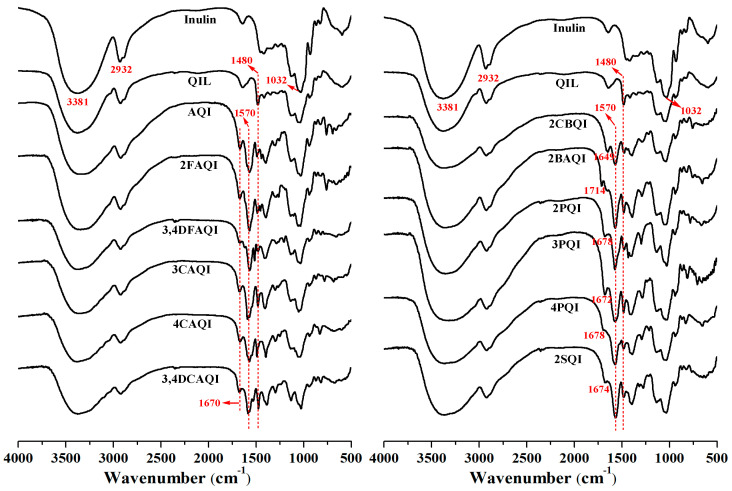
FTIR spectra of inulin, QIL, and QIL derivatives.

FTIR is a convenient and fast technique to observe the structural transformations that occur in a reaction. As shown in Figure 1, native inulin exhibited typical characteristic peaks of polysaccharide of O-H stretching vibration at 3381 cm^−1^, C-H stretching vibration at 2932 cm^−1^, and C-O-C stretching vibration at 1032 cm^−1^ [30]. After cationization, a sharp and strong peak appeared in the spectrum of QIL at 1480 cm^−1^, which was ascribed to the C-H symmetric bending of the trimethyl groups on the quaternary ammonium [20]. Additionally, a new peak at 1417 cm^−1^ was associated with the C-N stretching vibration. The data above indicated the successful synthesis of QIL. For the 12 kinds of QIL derivatives, the C-H symmetric bending of the trimethyl groups on the quaternary ammonium at 1480 cm^−1^ still existed. Additionally, new peaks appeared at around 1670 cm^−1^ and 1570 cm^−1^, corresponding to amide I and amide II absorption, respectively. These results provided evidence of the successful derivatization of QIL with aromatic amide.

**Figure 2:**
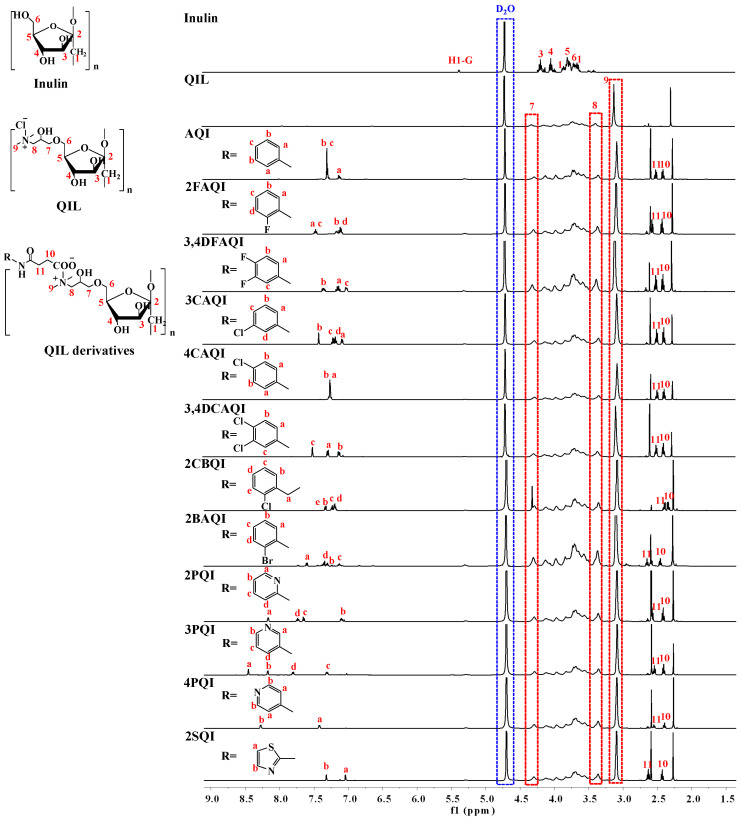
^1^H NMR spectra of inulin, QIL, and QIL derivatives, taking D_2_O as the solvent.

Figure 2 presents the ^1^H NMR spectra of inulin, QIL, and QIL derivatives. The hydrogen atom signals within the inulin backbone were detected within the chemical shift range of 3.40 to 5.35 ppm [27]. The ^1^H NMR spectrum of QIL exhibited new peaks at H7 (4.30 ppm), H8 (3.38 ppm), and H9 (3.10 ppm), which were attributed to the presence of CHPTAC [20]. These peaks persisted in the ^1^H NMR spectra of all the inulin derivatives, although they exhibited varying degrees of chemical shift due to the influence of neighboring functional groups. Furthermore, the ^1^H NMR spectra of QIL derivatives revealed additional signals in the range of 2.34–2.64 ppm, corresponding to H10 and H11. In addition, signals associated with phenyl, pyridyl, and thiazolyl groups were identified within the range of 6.98 to 8.16 ppm, with specific peaks indicated in Figure 2.

The aforementioned data corroborated the successful synthesis of QIL derivatives that incorporate aromatic amide functionalities.

### 3.2. Yields and DS Analysis

**Figure 3:**
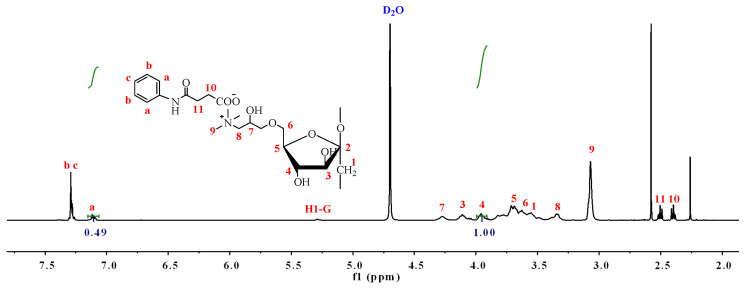
^1^H NMR spectrum of AQI and its integrals of hydrogen protons.

Table 1 summarized the yields and DS values of QIL and its derivatives. The DS values were calculated based on the ^1^H NMR spectra, with the integration of H4 proton serving as standard. For example, the DS of AQI was calculated as 0.49/1 = 0.49 in Figure 3, where 0.49 represented the integration values of Ha. Among all the derivatives, the DS of QIL reached 91.9%, demonstrating the high efficiency of the synthetic strategy used in this article. Among all the QIL derivatives, 3,4DCAQI exhibited the highest DS at 56.60%.

### 3.3. Antioxidant Activity

**Figure 4:**
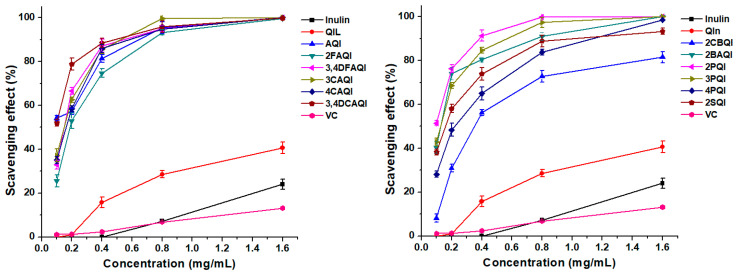
Hydroxyl radical scavenging activity of inulin, QIL, and QIL derivatives.

**Figure 5:**
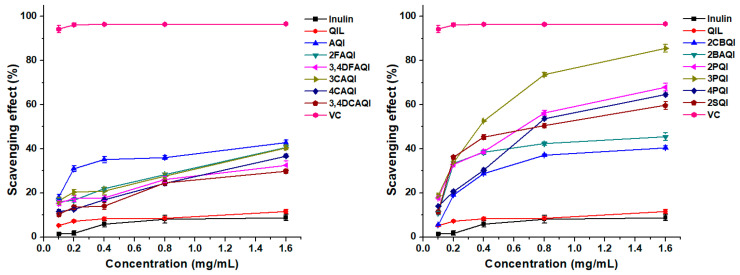
DPPH radical scavenging activity of inulin, QIL, and QIL derivatives.

**Figure 6:**
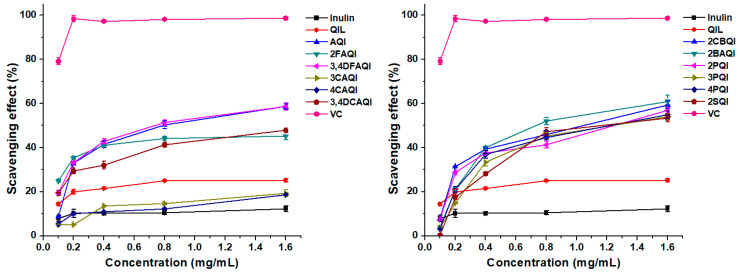
Superoxide radical scavenging activity of inulin, QIL, and QIL derivatives.

Antioxidants are vital components in human health products for regulating and preventing oxidative stress, and act as essential additives to prolong food and cosmetic stability [31,32]. The antioxidant properties of biomedical materials can reduce the oxidative stress generated after implantation, thereby lowering the risk of material-induced inflammatory responses and enhancing compatibility with host tissues. Simultaneously, materials with antioxidant activity can effectively mitigate oxidative damage, prevent cellular apoptosis, reduce tissue injury, and promote wound healing and tissue regeneration [33,34]. This study employed three distinct antioxidant assays to thoroughly evaluate the antioxidant capacity of inulin, QIL, and its derivatives, with key results illustrated in Figure 4, Figure 5 and Figure 6.

#### 3.3.1. Hydroxyl Radical Scavenging Activity

The hydroxyl radical is considered a highly hazardous free radical among reactive oxygen species (ROS), capable of interacting with various biomolecules such as proteins, DNA, and lipids, thereby leading to the development of numerous diseases [35]. According to the literature, some natural polysaccharides have exhibited potential as natural antioxidants [36]. In particular, inulin has been found to exhibit significant antioxidant activity at high concentrations because of the large amounts of hydroxyl groups [37]. As depicted in Figure 4, the scavenging rate of inulin was 24.02% at 1.6 mg/mL, which was slightly higher than that of VC. After quaternization, the scavenging rate of QIL increased to 40.69%. It was speculated that large amounts of positive charges of QIL play an important role in the process of stabilizing free radicals. Significantly, all the QIL derivatives showed greater hydroxyl radical scavenging activity than QIL. Most of the derivatives were able to completely scavenge hydroxyl radicals at a concentration of 1.6 mg/mL.

**Scheme 2:**
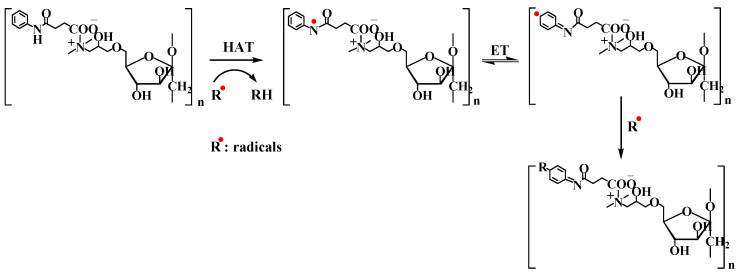
Free radical scavenging mechanism of AQI.

The scavenging process could be explained by hydrogen-atom-transfer (HAT) and electron transfer (ET) mechanisms as shown in Scheme 2. Reactive radicals first achieved a stable state by extracting hydrogen atoms from the N-H group of amides, generating active nitrogen center free radicals. These radical intermediates were stabilized by the resonance delocalization of the electrons within the aromatic ring [38]. According to theoretical research conducted by Kabanda [39], the combined evidence of charge reduction and suppressed spin density confirmed that Cu(II) was reduced to Cu(I) upon binding to CaD, while CaD itself was oxidized. This redox process stabilized the [CaD-Cu]^+^ complex, highlighting a metal–ligand co-oxidation mechanism. Cu^2+^ chelation preferentially occurred at the cooperative site formed by the carbonyl oxygen and the aromatic ring, accompanied by electron transfer from the ligand to the metal. The structures of the QIL derivatives in this article also contained a carbonyl oxygen and an aromatic ring. Therefore, these QIL derivatives were also likely to reduce the generation of free radicals by chelating metal ions, thereby exerting an antioxidant effect. Based on the above analysis, the high antioxidant activity of QIL derivatives was mainly attributed to the introduction of aromatic amide, which has been demonstrated as a crucial unit in other reported antioxidant compounds [40,41]. Moreover, the aromatic structure with special electronic effects was also a non-negligible factor in stabilizing free radicals.

#### 3.3.2. DPPH Radical Scavenging Activity

As shown in Figure 5, the scavenging activity of all test samples against DPPH radicals demonstrated enhancement as their concentrations increased. Inulin and QIL exhibited relatively low scavenging activity, with scavenging rate of 8.84% and 11.66% at the highest test concentration of 1.60 mg/mL, respectively, suggesting that quaternization had little effect on the DPPH radical scavenging activity of inulin. By contrast, the scavenging activity of QIL derivatives notably improved after the ion exchange reaction. It is well known that the hydrogen-donating ability of polysaccharide derivatives is a crucial factor influencing their radical scavenging activity [42]. The active hydrogen from the N-H group of amides plays an important role in stabilizing DPPH radicals. Thus, a significant improvement in DPPH radical scavenging activity was observed after incorporating aromatic amides. However, introducing halogen substituents into the benzene ring had little effect on enhancing the antioxidant activity of the QIL derivatives. When the influence of DS was considered, the derivatives without substituents on the benzene ring exhibited the strongest antioxidant effect. Among all the derivatives, 2PQI, 3PQI, 4PQI, and 2SQI, which contain aromatic heterocyclic structures, exhibited greater biological activity than the others. 3PQI had the strongest DPPH radical scavenging activity among all the derivatives, with scavenging rates of 18.86%, 33.90%, 52.61%, 73.62%, and 85.47% observed at concentrations of 0.1, 0.2, 0.4, 0.8, and 1.6 mg/mL, respectively. Here, the scavenging rates of these heterocyclic derivatives were correlated to their DS. The higher DS meant more active groups. It was reported that the heteroatoms (such as N, S, etc.) in aromatic heterocycles could influence the electron density of the aromatic ring through the involvement of their lone pair electrons, thereby altering their reactivity. In addition, aromatic heterocyclic compounds often possess a conjugated π-electron system, which allows them to effectively interact with oxygen radicals and donate electrons. By extending the length of the conjugated chain or introducing more conjugated structures, the antioxidant effect of the compound can be improved [43,44]. The results further proved that heterocyclic compounds with specific electronic effects, such as pyridine, imidazole, thiazole, and triazole, were effective in modifying polysaccharides to enhance bioactivity and design materials [45].

#### 3.3.3. Superoxide Radical Scavenging Activity

Figure 6 illustrates the superoxide radical scavenging activities of test samples. The positive control VC exhibited a strong ability to scavenge superoxide radicals, which could be completely eliminated even at low test concentrations. The scavenging ability of inulin was the weakest among all the samples, whose scavenging rate was only 12.29% at 1.6 mg/mL. After quaternization, the scavenging ability increased by about 10%. However, when aromatic amide was introduced to QIL, the scavenging abilities of 3CAQI and 4CAQI were reduced compared to QIL. On the contrary, other derivatives including AQI, 2FAQI, 3,4DFAQI, 3,4DCAQI, 2CBQI, 2BAQI, 2PQI, 3PQI, 4PQI, and 2SQI had notably improved scavenging abilities after ion exchange. Among them, 2BAQI had the strongest scavenging rate of 60.93%.

In summary, we concluded that the aromatic amide and quaternary ammonium salt can synergistically enhance the antioxidant activity of inulin. These derivatives achieved high antioxidant activity by means of HAT and ET mechanisms, and/or by chelating metal ions, especially showing significant effects in scavenging hydroxyl radicals. Hence, the series of inulin derivatives bearing quaternary ammonium salt and aromatic amide in this study are promising developed as antioxidant materials in biomedical materials, functional foods, and pharmaceutical applications.

### 3.4. Antibacterial Activity

Bacterial infection is one of the common complications following the implantation of biomedical materials, particularly in applications such as orthopedic implants and wound dressings. Therefore, imparting materials with antibacterial properties not only prevents infections but also promotes the healing process [1]. The synergistic effect of antibacterial and antioxidant properties plays a crucial role in enhancing material biocompatibility, promoting tissue repair, and reducing complications. By rationally designing and functionalizing biomedical materials with these dual capabilities, such multifunctional materials can improve therapeutic efficacy and further mitigate adverse reactions in clinical applications. Therefore, the antibacterial activity was also evaluated in this study.

As shown in Table 2, azithromycin exhibits a highly significant antibacterial effect. The MIC and MBC of azithromycin against *Escherichia coli* and *Staphylococcus aureus* were both 0.0078 mg/mL. In contrast, inulin showed no antibacterial activity at the test concentrations due to a lack of functional groups. However, QIL exhibited antibacterial activity after quaternary amination modification. Large amounts of positive charges play an important role in the antibacterial effects of QIL because they can alter the permeability of the cell wall, ultimately resulting in cell death. Moreover, QIL derivatives demonstrated enhanced antibacterial effects compared to QIL and exhibited a broad spectrum of action, being able to inhibit both Gram-positive and Gram-negative bacterial strains. The results indicate that the aromatic amide interact synergistically with quaternary ammonium salt to increase antibacterial activity. Notably, the derivatives modified with pyridine exhibited the strongest antibacterial activity among all the QIL derivatives. For instance, the MIC and MBC values of 3PQI against *Escherichia coli* were 0.125 and 0.25 mg/mL, while the MIC and MBC values against *Staphylococcus aureus* were 0.0625 and 0.125 mg/mL, respectively. When considering the influence of DS on antibacterial activity, 4PQI exhibits the most significant effect among all QIL derivatives. Increasing the DS of these derivatives would further enhance their antibacterial activity, bringing it closer to that of the positive control, azithromycin. Moreover, by comparing the MIC and MBC values of AQI, 2FAQI, and 3,4DFAQI, it is evident that the addition of halogen atoms can enhance antibacterial activity, and the presence of more halogen atoms results in stronger antibacterial activity. Similar findings were noted when evaluating 3CAQI, 4CAQI, and 3,4DCAQI.

**Figure 7:**
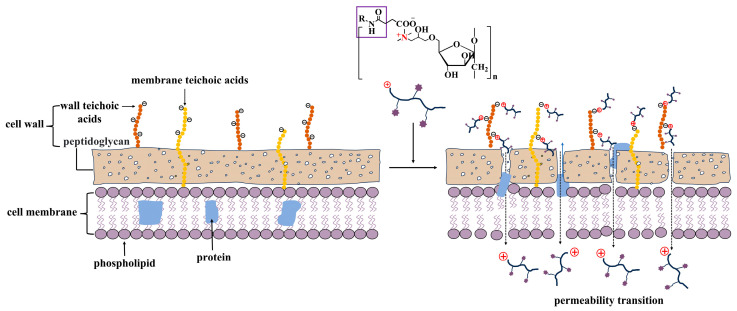
Antibacterial mechanism of QIL derivatives against Gram-positive bacteria.

Through further analysis, it was found that derivatives have a more significant inhibitory effect on Gram-positive bacteria, *Staphylococcus aureus*. The possible mechanism was shown in Figure 7. The Gram-positive bacteria contain wall teichoic and membrane teichoic acids negatively charged [46], which could establish interactions with the positive charges of quaternary ammonium salt. This leads to a large accumulation of quaternary ammonium salts on the surface of bacteria, which disrupts the cell wall structure, causing the leakage of intracellular substances and ultimately leading to bacterial death. In addition, the quaternary ammonium salts in the QIL derivatives serve as a bridge, attracting more amounts of aromatic amide to interact with the bacterial surface, thereby improving the antibacterial effect.

In comparison, the cytoderm structures of Gram-negative bacteria exhibit greater complexity in chemical composition and structural organization when compared to Gram-positive bacteria. Gram-negative bacteria possess two layers outside the cytoplasmic membrane, resulting in a more formidable physical barrier [47,48]. Such complicated structure may account for the weaker inhibitory effect of QIL derivatives on Gram-negative bacteria. Therefore, it was concluded that the chemical modifications of inulin with quaternary ammonium salt and aromatic amide synergistically lead to an increase in antibacterial activity. Further studies are needed to investigate the specific antibacterial mechanism of these derivatives to develop new broad-spectrum antibacterial agents.

### 3.5. Cytotoxicity

**Figure 8:**
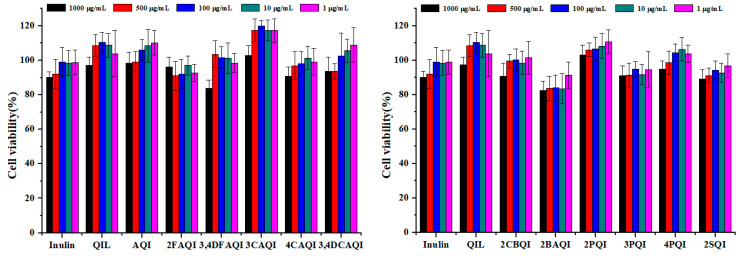
Cytotoxicities of inulin, QIL, and QIL derivatives on L929 cell lines according to the CCK-8 test.

Cytotoxicity assays can help assess the safety of the prepared samples as biomaterials and their use in contact with human tissues. The results are shown in Figure 8. The safety of inulin has been verified by the Food and Drug Administration (FDA) in 2018. This study arrived at the same conclusion, as the survival rate of L929 cells exposed to inulin reached 90%. The cell viabilities of QIL are higher than inulin. In addition, all the QIL derivatives did not show significant cytotoxicity. The findings suggested that inulin have the potential to establish a conducive environment for the controlled release of small molecule pharmaceuticals, indicating their viability for application in drug delivery systems.

## 4. Conclusions

In this study, quaternized inulin (QIL) was first synthesized and then modified with series aromatic amides. Their structures were confirmed by FTIR and ^1^H NMR spectroscopy. The degree of substitution (DS) was calculated to be between 13.1% and 56.6% based on the ^1^H NMR spectra. The synthesized QIL derivatives exhibited high antioxidant activity, particularly showing significant effects in scavenging hydroxyl radicals, with scavenging rates reaching 100% at 1.6 mg/mL. The principal antioxidant mechanisms discussed in this paper include hydrogen atom transfer (HAT) and electron transfer (ET) mechanisms, as well as metal ion chelation mechanisms. The chemical modifications of inulin with quaternary ammonium salt and aromatic amide synergistically lead to an increase in antibacterial activity. QIL derivatives exhibited a broad spectrum of action against both Gram-positive and Gram-negative bacterial strains, and they were more efficient against Gram-positive bacteria (*Staphylococcus aureus*). Through further analysis of the structure–activity relationship, we concluded that the addition of halogen atoms could enhance the antibacterial activity, and the presence of more halogen atoms results in stronger antibacterial activity. Furthermore, the cytotoxicity of the QIL derivatives on L929 cells was evaluated by the CCK-8 method. All the derivatives showed no toxicity, as their cell survival rates exceeded 80%.

This study systematically evaluated the biological effects of QIL derivatives bearing aromatic amide. Inulin derivatives integrating antioxidant activity, antibacterial properties, and non-toxicity exhibit capabilities to mitigate oxidative damage and inhibit infections, demonstrating promising application prospects in biomedical materials, functional foods, and pharmaceutical applications.

## Figures and Tables

**Table 1:** The yield and DS of QIL and its derivatives.

Compounds	Yields (%)	DS (%)
QIL	84.22	91.90
AQI	53.18	24.60
2FAQI	64.49	39.85
3,4DFAQI	61.72	32.90
3CAQI	60.77	53.10
4CAQI	67.34	51.35
3,4DCAQI	62.18	56.60
2CBQI	61.43	30.45
2BAQI	60.52	29.60
2PQI	58.44	28.40
3PQI	57.38	30.30
4PQI	57.15	13.10
2SQI	57.47	25.10

**Table 2:** The MIC and MBC values of inulin, QIL, and QIL derivatives against *Escherichia coli* and *Staphylococcus aureus*.

Compounds	*Escherichia coli*		*Staphylococcus aureus*	
	MIC (mg/mL)	MBC (mg/mL)	MIC (mg/mL)	MBC (mg/mL)
Inulin	>16	>16	>16	>16
QIL	8	16	8	8
AQI	4	8	2	4
2FAQI	1	2	1	2
3,4DFAQI	0.25	0.5	0.125	0.125
3CAQI	4	8	0.5	0.5
4CAQI	4	8	0.5	1
3,4DCAQI	0.25	0.5	0.125	0.25
2CBQI	2	4	2	4
2BAQI	1	2	1	2
2PQI	0.125	0.5	0.125	0.25
3PQI	0.125	0.25	0.0625	0.125
4PQI	0.125	0.25	0.125	0.125
2SQI	0.25	0.5	1	2
Azithromycin	0.0078	0.0078	0.0078	0.0078

## Data Availability

The data presented in this study are available within the article.

## Abbreviations

The following abbreviations are used in this manuscript:

CCK-8	cell counting kit-8
DP	degree of polymerization
CHPTAC	(3-chloro-2-hydroxypropyl) trimethylammonium chloride
QIL	quaternized inulin
EDTA-2Na	disodium ethylenediaminetetraacetate
NBT	nitrotetrazolium blue chloride
Tris	tris(hydroxymethyl)aminomethane
PMS	phenazine methosulfate
NADH	nicotinamide adenine dinucleotide
DPPH	2,2-diphenyl-1-picrylhydrazyl
CDI	carbonyldiimidazole
DMSO	dimethyl sulfoxide
RT	room temperature
MWCO	molecular weight cutoff
FTIR	Fourier-transform infrared
TMS	tetramethyl silane
NMR	nuclear magnetic resonance
AFU	anhydrofructose units
VC	Vitamin C
MIC	minimum inhibitory concentration
MBC	minimum biocidal concentration
ROS	reactive oxygen species
HAT	hydrogen-atom-transfer
ET	electron transfer
FDA	Food and Drug Administration
RPMI	Roswell Park Memorial Institute
